# The Radical Operation as Applied to Middle Ear Diseases1Read before the Section on Ophthalmology, Laryngology and Otology, Buffalo Academy of Medicine, February 12, 1900.

**Published:** 1900-04

**Authors:** George F. Cott

**Affiliations:** Buffalo, N. Y.


### THE RADICAL OPERATION AS APPLIED TO MIDDLE EAR DISEASES.[1]

By GEORGE F. COTT, M. D., Buffalo, N. Y.

THIS paper may be considered a supplement to the one brought before the Pathological Section of the Academy in Dec., 1899. At that time the pathological conditions which indicated radical treatment were considered. I will briefly allude to the indications again with other important points which necessarily go with the operation. As early as 1889, Koerner drained the middle-ear cavity by removing bone obstructing a free flow of pus; likewise Kuester, Stacke, Zaufal, Urbantschitsch, McEwen and others. The latter, however, operated chiefly upon cases which suffered rather from results of negected middle-ear disease. At least three of those mentioned have already completed their hundredth case with phenomenal success. During the last ten years progress has been slow, the number of operators not increasing very rapidly. Of course, we have to deal with a pus cavity, which can easily cause infection of the meninges or the brain itself. The brain may also be injured by a chisel or drill, and so may the large bloodvessels and the facial canal. The serious nature of the operation, therefore, keeps the otological surgeon from venturing.

We promise nothing to the patient, however, except the removal of dead bone and proper drainage. If such cases are left alone one of many complications may occur, and these must also be explained to patient or parents. Cases coming under observation are usually due to scarlet fever, measles, or diphtheria infection; occasionally, caries is found in strumous children.

Most of the chronic cases are due to neglect. For centuries no remedies were known; but since the advent of antiseptic surgery the masters put their best energies at work to keep this branch up with general surgical progress.

Chronic middle-ear suppuration is invariably due to the acute process and occurs mostly in early childhood. Very few cases occur after the age of thirty.

The first more or less radical effort at removal of dead bone and making a large canal for drainage was introduced by Schwartze nearly thirty years ago, and for two decades his method was the only one in vogue. Many cases, however, did not recover, since the operation did not reach the parts affected, but allowed the diseased bone to

1. Read before the Section on Ophthalmology, Laryngology and Otology, Buffalo Academy of Medicine, February 12, 1900.

continue its destructive work. After twenty years of Schwartze gradual modifications were introduced by a number of German-speaking otologists, who from time to time published their results. Stacke's work being among the first to be published, "the name of Stacke's operation" was applied to his modification. Stacke, however, has fallen in line with other gentlemen in the designation of "radical" since no man can lay claim to priority, each one having contributed his mite to its final perfection. The operation as it now stands comprises opening of the mastoid, which if diseased is cleaned out, then follows opening of the antrum, then removal of the external wall of the attic.

Some operators remove the remains of the drumhead first, then the ossicles which is the more rational. This procedure converts the entire diseased parts into one cavity. This, after curetting, admits the parts to be thoroughly drained and finally filled up with granulation tissue.

*Indications.*—Simple opening of the mastoid process, according to Schwatze, is insufficient, drainage being incomplete. If, however, this has been done without result further operative measures must be undertaken. Removal by drill, chisel or curette is necessary. This cannot be done through the natural opening, unless the carious process is limited to the ossicles, and the inflammatory product has not caused too much thickening of the canal walls, precluding manipulation through the meatus.

When suppuration is chronic and dead bone cannot be positively made out and treatment has been extended over a certain period without success, radical interference is indicated. Second, retained pus, if its cause cannot be removed within a reasonable length of time; third, hyperostosis which prevents proper drainage, examination and treatment; fourth, when intracranial trouble manifests itself during chronic suppuration; fifth, labyrinthine involvement, or if the facial canal has become affected.

Every chronic suppurative process does not call for radical interference, since discharge from the middle-ear may last for ten or twenty years, in some cases, without the presence of caries. Such cases usually get well with ordinary treatment. It may be impossible to make a proper diagnosis at first, but the results of treatment will prove that the disease does not extend beyond the mucous membrane. Occasionally carious ossicles require removal, when recovery takes place. It does not follow that other parts of the tympanum and adjacent cavities are affected. Any number of cases may be cited showing recovery after ossiculectomy. When, however, the antrum is affected, removal of the ossicles may mitigate suppuration, but a complete cure can rarely be effected. Schwartze's mastoid operation can accomplish very little when the walls of the middle-ear, the antrum, external canal and ossicles are involved, for the reason that sclerosis of the entire petrous portion of the temporal bone is the rule, with occasional exceptions.

Now, what is accomplished by the so-called radical operation? You all know many cases of incurable middle-ear disease; in other words, chronic discharge from the ear. The patients have been taught it dangerous to interfere, and this became so notorious that treatment was often not permitted. Let us see what benefit the so-called incurable cases may derive: (1) We open into the antrum through a sclerosed mastoid, although the antrum may be very small, and thereby produce direct drainage without injuring the labyrinth or aqueductus fallopii; (2) we readily enter the desired recesses, even though the sigmoid sinus encroaches upon the mastoid out of the ordinary, without especial harm to the sinus or its contents; (3) we open up all the diseased spaces, from the cells to the middle-ear, thereby creating a funnel-shaped cavity with the large end pointing externally, enabling the surgeon not only to remove all dead bone, but also to observe the future progress of healing; (4) we so constitute the opening that future interference becomes easy and recurrence readily dealt with; (5) last, but not least, hearing is generally improved, provided the internal ear is not involved. Now, this is not accomplished by conveying sound waves to the oval window where the stapes had been attached, but by the sound waves acting upon the tissue filling the cavity made by the operator and are conveyed to the membrana tympani secondaria or the round window. This membrane seems to escape in a measure the destructive inflammatory action, so as still to remain sensitive to sound waves and transmit them to the labyrinthine fluid. This conclusion I infer from the necessary thickening of the covering of the oval window, when the footplate of the stapes has suffered with the destruction of the ossicles and it thereby became unimpressionable. It, therefore, seems to me quite a reasonable deduction.

It is conceded by the best of operators in otology that the radical operation is one of the most responsible in the whole domain of surgery. I believe this, because there are at present very few who undertake it.

Why is it so formidable an operation? What are we liable to encounter? In opening the mastoid process the sigmoid sinus is easily penetrated. There may have been so much destruction of bone that while seeking the antrum, which may be very small, the base of the brain may be injured, and dealing with a pus cavity the infection may readily be conveyed to the meninges or the brain itself, causing abscess. The semicircular canals of the internal ear may be opened. In removing the external wall of the attic a slip of the chisel may cause fracture of the external labyrinthine wall. A thin plate of bone, separating the internal carotid artery from the anterior part of the middle-ear may be penetrated, causing fatal hemorrhage. The partition separating it from the tympanum may be softened or carious and therefore easily penetrated. I have found sixteen cases recorded where fatal hemorrhage took place through contiguous inflammatory softening. Likewise, the floor of the middle-ear, which separates that cavity from the bulb of the jugular vein may be injured carelessly. The glenoid cavity may be opened, infecting the joint of the lower jaw. The upper part of the parotid gland may be laid bare by the removal of the external cartilage; by chiseling too low, the facial nerve will surely be injured. Laying bare the facial nerve is an accident which sometimes cannot be avoided, when its position is out of the ordinary. It usually courses forward about one inch from the external opening, but has been found only one half inch from that orifice.

Some of these presumed accidents cannot be avoided, while others must be. Those parts unavoidably injured depend for their successful issue entirely upon the strictest kind of antiseptic treatment, not only during the operation, but also at redressings. After the different nooks and corners have been converted into one cavity, all shreds still remaining are cleaned out, the bone curetted and smoothed over, then a probe is used in seeking for sinuses leading from the external canal or middle-ear and, if any are found, they must be carefully enlarged for drainage, no matter where they may be situated. In one case of Koerner's a semicircular canal partially scraped out, a sinus having been found leading into it, healed kindly without any untoward result. If a sinus be found leading into the cranial cavity it must be treated likewise; if neglected fatal meningitis or abscess may follow and all our efforts have been in vain.

Case I.—Mrs. T., of Olean, N.Y., aged 29 years; discharge from left ear twenty-one years, caused by an attack of measles at the age of eight. Could hear watch upon contact only. Could not hear whispering voice at all. Symptoms mitigated by constant treatment, but discharge never ceased entirely. Operation April 26, 1899, assisted by Dr. Blaauw, at Riverside Hospital, Buffalo. Found bone sclerosed throughout and the mastoid cells were almost entirely absent. The tympanic cavity contained the head of the malleus only. After converting all the cavities into one large funnel shaped space, by two hours of chiselling and scraping away of sclerosed bone the auricle was replaced and the cavity drained posteriorly with iodoform gauze. The external auditory canal was prevented from collapsing by a rubber tube; through which drainage also took place. In this case there was no reaction; the temperature never rose above 99½°; pulse ranged from 90 to 120; slept four hours first night and five hours the next. The only pain she had at any time was caused by the tube in the ear and the bandage. On May 22d, four weeks after the operation she heard the watch eight inches and whispering voice two feet. February 9th, she writes me she can hear a watch six inches and the hearing also improved for whispering voice. She has now, ten months after the operation, a slight watery discharge, however not like pus and her general health is very much improved. This patient sat up in bed the third day and left the hospital on the eighth.

Case II.—Miss Eleanor T, aged 21, Lockport, N. Y., had earache a long time, previous to an attack of measles at the age of eight, when her left ear began to discharge which continued for twelve years. Could not hear the watch tick upon contact, or the clock in her room, nor whispering voice. Two years ago I removed some granulation tissue and curetted the posterior wall of the external extremity of the canal. There some denuded bone was found. This treatment mitigated the discharge very much, and held it pretty well in check while the after-treatment lasted. July 12, 1899, the radical operation was performed at the Riverside Hospital, assisted by Dr. Charters and Dr. King. The cavity was dressed the fourth day and patient allowed to leave the hospital. In this case there was some discharge for three months after the operation. This patient had very severe headache almost every day, especially on the top of the head. Since the operation she has had practically none. Weight before the operation, 98 lbs.; today, 109 lbs. February 12, hearing after seven months, watch, ten inches; whispering voice, seven feet; acoumeter, eight feet; ordinary conversational voice, thirty feet; no drainage.

Case III.—Little boy, three years old. During an attack of a severe type of scarlet fever had both middle ears affected, followed by suppuration. Child was in a very bad condition and emaciated. It was decided from the symptoms that the mastoid was affected, which was opened, but found comparatively healthy. On further examination with the probe, the canal was found denuded, the soft parts were dug out, and the whole cavity cleaned out. Nothing of the ossicles could be found. This case was dressed by Dr. J. H. Potter, of Black Rock, for several months. The discharge never entirely ceased. A second operation was undertaken seven months after the first. The ear was dug out again, granulation tissue removed, carious walls curetted again, nutrition being so poor that the parts would not heal properly. At the second operation a piece of the skull was removed, about three-quarters of an inch in diameter, above and behind the mastoid process, laying bare the dura mater. The scalp was loosened for several inches and brought together and stitched with silkworm-gut. The parts healed kindly, and now after a lapse of several months very slight discharge remains.

Case IV.—Little girl, aged 5; referred to me by Dr. Auel, for chronic discharge of right ear. A polyp was removed and discharge ceased for several months, when it again appeared and continued for two years. The external auditory canal was so narrowed from inflammatory thickening and was so painful, that it was impossible to make a proper examination. Operation at Riverside Hospital, assisted by Dr. Cruickshank and Dr. King, February 1, 1900. The middle-ear cavity contained nothing but shreds of the tympanic membrane. The petrous bone and mastoid process were entirely sclerosed. Operation took an hour and a half, but the child was under the influence of chloroform for over three hours. No especial reaction occurred, and the child slept several hours during the night. Temperature never rose above 100°, but twice during the first week, pulse ran up to 150; after a movement of the bowels it came down to 120. In a week, after the tampon in the external ear had become saturated by the exudate, the dressings were removed. At the second dressing, three days later, the cavity contained a very slight, healthy discharge. In this case there was no drainage behind the ear. The child suffered no pain at any time, excepting upon movement of the lower jaw, the joint having been disturbed while removing the cartilage. Hearing power had improved in this case to watch, four inches; acoumeter, four feet; loud voice, ten feet; and whispering, five feet.

531 Mooney-Brisbane Building.

---

